# MEHP interferes with mitochondrial functions and homeostasis in skeletal muscle cells

**DOI:** 10.1042/BSR20194404

**Published:** 2020-04-17

**Authors:** Yi-Huan Chen, Yi-Ju Wu, Wei-Cheng Chen, Tzong-Shyuan Lee, Tsui-Chun Tsou, Hsuan-Chia Chang, Sheng-Wen Lo, Shen-Liang Chen

**Affiliations:** 1 Department of Life Sciences, National Central University, Taoyuan, Taiwan; 2 Graduate Institute of Physiology, National Taiwan University, Taipei, Taiwan; 3 National Institute of Environmental Health Sciences, NHRI, Miaoli, Taiwan

**Keywords:** DEHP, MEHP, metabolism, mitochondria, muscle, OXPHOS

## Abstract

Di (2-ethylhexyl) phthalate (DEHP) is a plasticizer frequently leached out from polyvinyl chloride (PVC) products and is quickly metabolized to its monoester equivalent mono(2-ethylhexyl) phthalate (MEHP) once enters organisms. Exposure to DEHP/MEHP through food chain intake has been shown to modified metabolism but its effect on the development of metabolic myopathy of skeletal muscle (SKM) has not been revealed so far. Here, we found that MEHP repressed myogenic terminal differentiation of proliferating myoblasts (PMB) and confluent myoblasts (CMB) but had weak effect on this process once it had been initiated. The transition of mitochondria (MITO) morphology from high efficient filamentary network to low efficient vesicles was triggered by MEHP, implying its negative effects on MITO functions. The impaired MITO functions was further demonstrated by reduced MITO DNA (mtDNA) level and SDH enzyme activity as well as highly increased reactive oxygen species (ROS) in cells after MEHP treatment. The expression of metabolic genes, including *PDK4, CPT1b, UCP2, and HO1*, was highly increased by MEHP and the promoters of *PDK4* and *CPT1b* were also activated by MEHP. Additionally, the stability of some subunits in the oxidative phosphorylation system (OXPHOS) complexes was found to be reduced by MEHP, implying defective oxidative metabolism in MITO and which was confirmed by repressed palmitic acid oxidation in MEHP-treated cells. Besides, MEHP also blocked insulin-induced glucose uptake. Taken together, our results suggest that MEHP is inhibitory to myogenesis and is harmful to MITO functions in SKM, so its exposure should be avoided or limited.

## Introduction

Plastic wares/tubing are widely used in our daily life so plasticizers released from these products will inevitably enter our body via various pathways/routes. Many of these plasticizers act as endocrine disrupters and have been linked to the rise of metabolic disorders. DEHP is a plasticizer included in the polyvinyl chloride (PVC) production process to increase the flexibility, transparency, and durability of PVC plastics. Unfortunately, as DEHP is not covalently linked to the polymers, it is easily leached out from these PVC products and thus people are continuously exposed to this compound due to our daily contact with plastic products. As a result, exposure to DEHP has become virtually continuous and essentially unavoidable, a fact that is highlighted by human biomonitoring studies [1,2].

Once entered human body, DEHP is quickly metabolized to its monoester equivalent mono-(2-ethylhexy) phthalate (MEHP) by lipase through cleaving a side chain from DEHP. MEHP is preferentially absorbed into circulation and therefore its biological effects on human health are hence of major concern [3]. The exposure of general human population to DEHP/MEHP ranges from 3 to 30 µg/kg/day, but the systemic concentration of DEHP/MEHP in patients under intensive care can be increased to the level of 8.5 mg/kg/day due to the leaching from medical wares and tubes. Furthermore, blood stored in PVC based bag for a certain period can also contain high concentration (about 2.5 mg/dl or 64 µM) of DEHP/MEHP leached out from the bag. These observations raises the concern that this high level DEHP/MEHP might aggravate the conditions of these patients under intensive care [4–7]. In addition to affecting endocrine system, they also affect metabolism in various other pathways [8].

Previous studies have shown that DEHP and its metabolic derivatives can serve as xenobiotic proliferators of the organelle peroxisome [9], which is involved in various metabolic pathways, including β-oxidation of fatty acids (FA) and detoxification of hydrogen peroxide [10]. DEHP/MEHP promotes the proliferation of peroxisomes by functioning as the ligands of the nuclear hormone receptors called peroxisome proliferator receptors (PPARs), especially the major PPAR isoform, PPARα, in liver. DEHP/MEHP preferentially activates PPARα activity but poorly on that of PPARβ/δ and PPARγ [9]. The physiological implications of DEHP triggered peroxisomes proliferation are manifested by the increased fatty acid utilization and β-oxidation, but reduced utilization of glucose; therefore, subjects exposed to DEHP show reduced fatty acids, triacylglycerol, but increased glucose levels in the blood, while pyruvate and lactate are accumulated in the SKM [11]. Furthermore, the insulin-induced glucose uptake is partially impaired in DEHP-exposed subjects, implying that DEHP might either induce or aggravate the insulin resistance syndrome in patients with Type 2 diabetes [12]. Interestingly, the effects of DEHP on cellular metabolism cannot be fully recapitulated by the nature or specific ligands of PPARs [13].

Skeletal muscle (SKM) constitutes approximately 40% of the adult body mass and is the major site of glucose metabolism, fatty acid oxidation, and cholesterol efflux; therefore, proper regulation of SKM metabolism is critical to the functional adaptation of SKM and the integrity of the systemic/whole body metabolism [14]. DEHP/MEHP is expected to have profound effects on SKM metabolism as PPARs are highly expressed there and their ligands induced strong fatty acid β-oxidation in SKM [15–18]. Due to the important role of SKM in systemic metabolism, it is of interest to examine the effects of DEHP/MEHP on SKM metabolism and discuss how this might contribute to systemic metabolic disorders/syndromes, especially insulin resistance, Type 2 diabetes, and obesity.

SKM is an organ facing continuous cycle of damage and regeneration, and it contains high level of stem cells, called satellite cells, for regenerating damaged myocytes. Activated satellite cells proliferate and differentiate into myoblasts that eventually fuse with each other to form multinucleated giant cells called myotubes [19]. This myogenic process is regulated by a group of bHLH-motif containing transcription factors (including Myf5, MyoD, Myogenin, and Mrf4) that are collectively as myogenic regulatory factors (MRFs) [20]. Whether DEHP/MEHP has any effect on the recruitment and differentiation of satellite cells is an interesting open question that needs to be further addressed. Besides, the effects on MRFs function will also be clarified.

Mitochondria and peroxisomes are the major organelles involved in the cellular oxidative metabolism and they collaborate in many metabolic processes, especially fatty acid β-oxidation (FABO) and ROS metabolism [21]. This intimate relationship suggests that, when peroxisomes are regulated by DEHP/MEHP via PPARs in other tissues, both organelles might be similarly regulated in SKM, either directly or indirectly to achieve oxidative metabolism collaboratively. Mitochondria are the power houses of eukaryotic cells and they provide ATP currency through oxidative phosphorylation (OXPHOS) of reducing equivalents [22], so the functions of mitochondria are highly demanded in the high energy requiring SKM. To date, the effects of DEHP/MEHP on mitochondria in SKM are relatively less known so more endeavor is invited to investigate how their functions and homeostasis are influenced by these plasticizers.

## Materials and methods

### Plasmids

The reporters driven by promoters of *PDK4, CTP1b*, and *M-cadherin*, respectively, have been described in our previous studies [23,24]. The promoter of *Ndufb8* (+179∼-2471) was amplified from mouse genomic DNA by PCR (25 cycles) and cloned into the *Xho*I site of pStable-luc vector. The *MTS-RFP* coding sequence was amplified from the plasmid pMITO-RFP-GFP and inserted into the *EcoR*V site of the pCDNA3.1 vector. The primer sequences for amplifying *Ndufb8* promoter and *MTS-RFP* are shown in Supplementary Table S2.

### Cell culture and transient promoter activity analysis

C2C12 myoblasts were incubated at 37°C in a humidified 5% CO_2_ atmosphere. To maintain the ability of proliferation, cells were cultured in DMEM supplemented with 20% FCS and stimulated to differentiate by replacing with differentiation medium (DM) containing DMEM supplemented with 2% horse serum. The medium was changed every 2 days, and the myotube (MT) was examined after 4 days in DM (DM4).

For transient promoter assay, reporters, in which luciferase expression was driven by promoters from the genes of interest, were transfected into C2C12 myoblasts using the T-Pro NTR-II transfection reagent (T-Pro Biotechnology) for overnight before changed to differentiation medium and incubated for 48 h. Then, cells were harvested and the promoter activity was tested using the luciferin (VivoGlo Luciferin, Promega) mixture (20 mM Tricine, 2.67 mM MgSO_4_, 1.07 mM (MgCO_3_)_4_. Mg (OH)_2_·5H_2_O, 0.1 mM EDTA, 33.3 mM DTT, 270 μM Coenzyme A, 530 μM ATP, 470 μM luciferin) with a Clarity 2 luminometer (BioTEK; Winooski, VM). All experiments were performed in triplicates and repeated at least three times.

### Cell viability assay

C2C12 cells were seeded and then treated with different dosages of MEHP for 2 days. Cell viability was detected using an MTT assay (Sigma-Aldrich). The reaction product was measured by spectrophotometer with absorbance at wavelength 570 nm.

### Immunofluorescence staining

C2C12 cells were cultured in six-well plates and then treated with 100 μM MEHP at PMB, CMB, and DM3 stage. DM3 indicated that cells were stimulated into differentiation for 3 days. Cells in all treatments were harvested after in the differentiation medium for 5 days. Then, they were washed extensively by PBS before fixed in 4% paraformaldehyde for 15–30 min, and blocked in blocking solution (0.2% Fish Skin Gelatin and 0.2% BSA in PBS) for 30 min. Then, cells were incubated with anti-MHC (clone 32, Sigma) at 4°C overnight and followed by incubation in Alexa Fluor® 568 secondary antibody (in blocking solution) at room template for 1 h. To visualize the nuclei, cells were stained by 100 ng/ml DAPI for 10 min. Samples were mounted and images were viewed and analyzed by Carl Zeiss Axio Observer A1 fluorescence microscope with Axio Vision software.

### Quantitative RT-PCR (qRT-PCR)

The detailed protocol of qRT-PCR has been described in our previous works [24,25]. Briefly, myotubes incubated in differentiation for 3 days (DM3) were treated with DMSO or 100 μM MEHP for 2 days. Then, total RNA was extracted and cDNA was synthesized by the Superscript III kit (Invitrogen) according to the manufacturer’s protocol. The qPCR product was detected by SYBR Green reaction mix (Power SYBR Green PCR master mix, Applied Biosystems). All reactions were performed in ABI 7300 sequence detection system with an amplification program of 40 cycles. The qRT-PCR primer sequences used in this study were described in Supplementary Table S1.

### Determine the DNA level of mitochondria

C2C12 cells were lysed and incubated with tail buffer (1% SDS, 0.1 M NaCl, 0.1 M EDTA, and 0.05 M pH8.0 Tris-HCl) and 10 mg/ml proteinase K at 56 °C for 1 h. About 0.5 M EDTA (pH 8.0) was used to reduce the activity of DNase. After centrifugation at 4°C, the supernatant was mixed with 99% EtOH and shaken gently to precipitate genomic DNA. Genomic DNA was washed by 99% EtOH and then dissolved by TE buffer (10 mM pH 8.0 Tris-HCl with 1 mM EDTA). Genomic DNA was purified by phenol/chloroform mixture and then precipitated by isopropanol. After washed with 75% EtOH, genomic DNA was dissolved by TE buffer. The ratio of mitochondrial DNA and nuclear DNA (mtDNA/ncDNA) was determined by qPCR (40 cycles) and used to determine the content of mitochondria after MEHP treatment. *Cytochrome-b* was presented as the marker of mtDNA and *MyoD* was presented as ncDNA. The experiment was analyzed by qPCR (with a program of 40 amplification cycles) and the primer sequences were shown in Supplementary Table S1.

### Mitochondrion functional assays

C2C12 cells were trypsined and then were lysed with sonication in PBS. After centrifugation, 50 μg of total protein was used for the enzymatic assays. The SDH assay was performed as previously described [26]. Briefly, 40 mM sodium succinate (pH > 7), 8 mM sodium azide, 0.005% 2,6-dini-trpphenolindophenol (DCPIP), and cell lysate were incubated at 37°C for 30 min. The reaction product was examined by reading the absorbance at 600 nm in a spectrophotometer (GeneQuant 100, GE). The level of SOD was determined using a SOD assay-WST kit (19160-1 KT, Sigma). Cell lysate was mixed with WST and enzyme solution before incubated at 37°C for 20 min. The reaction product was analyzed by spectrophotometry with absorbance at 450 nm. The SOD activity in the lysate was calculated by the percentage of reduction in the 450 nm absorbance.

### Determination of ROS levels

The detailed protocols for these assays have been described in our previous study [24]. Briefly, cells were incubated in KRPH buffer containing 30 μM H2-DCFDA at 37°C for 30 min. Then, cells were extensive washed with PBS before trypsinzed (with 0.25× trypsin) and re-suspended in PBS buffer. The fluorescence of H2-DCFDA in the cells was measured in a fluorescence spectrophotometer (Hitachi F-4500) with excitation and emission at 495 and 529 nm, respectively. The fluorescence was normalized with protein level after lysis.

### Western blot analysis

C2C12 cells were suspended in RIPA buffer and protease inhibitors. After centrifuging, protein concentration was determined by Pierce BCA Protein Assay (ThermoFisher). Total protein (50 μg) was resolved on SDS-PAGE gel, and then transferred onto PVDF membrane (Pall FluoroTrans W membrane, PALL). After blocking with blocking buffer (PBS containing 0.5% Tween 20 and 5% milk), the membrane was hybridized by primary antibodies against MHC (MY-32, Sigma-Aldrich), OXPHOS (ab110413), and Gapdh (ab9482, Abcam) at 4°C overnight. HRP-conjugated secondary antibodies were added into the membrane and incubated at room temperature for 1 h. After washed with PBS, the HRP signal was detected by an enhanced chemiluminescence kit (GTX400006, Genetex) and viewed with X-ray film.

### Fatty acid oxidation

This assay measures the β-oxidative activity of mitochondria by quantitation of ^3^H-labeled H_2_O generated at the acyl-CoA dehydrogenase step [23]. First, C2C12 cells were incubated in α-MEM with 2 μCi BSA conjugated ^3^H-labeled palmitic acid (NET043001MC, Perkin Elmer) and 50 μM palmitic acid at 37°C for 5 h. Then, the un-metabolized ^3^H-palmitic acid in the conditioned medium was removed by phenol/chloroform. The ^3^H activity in the aqueous phase was detected by a scintillation β-counter (LS6500, Beckman).

### Mitochondrial membrane potential level

Cells grown on six-well plates were washed with PBS thoroughly before incubated with KRPH buffer containing JC-1 (2 µM; #1130-5, BioVision) for 30 min. Then cells were trypsinized and re-suspended in PBS. Cells were transferred to a 2 ml plastic cuvette and their emission of light at 590 (aggregates) and 530 (monomers) nm wavelengths after excited with light of 488 nm wavelength was determined using the F-4500 fluorescence spectrophotometer (Hitachi). Some cells were treated with JC-1 and Carbonyl cyanide-4-(trifluoromethoxy)phenylhydrazone (FCCP, 25 µM) at the same time to test the reliability of JC-1 staining.

### Statistical analysis

All experiments were performed at least three times with similar results observed. The *t*-test was used to analyze the differences between the two groups. *P*-value < 0.05 was considered statistically significant.

## Results

### High dose MEHP is toxic to myoblasts

**Figure 1:**
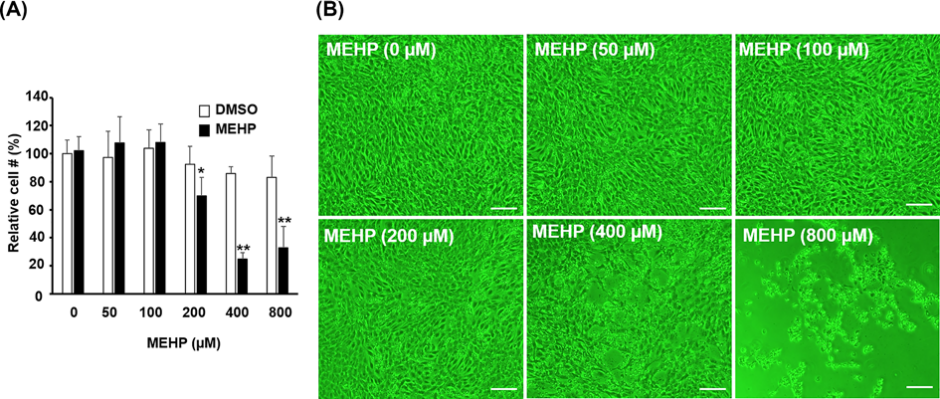
The cell proliferation effects of MEHP C2C12 myoblasts at CMB stage were treated with various doses of MEHP for 2 days, then, cell viability was examined by MTT assay (**A**). The vehicle (DMSO) volume varied from 0, 0.5, 1, 2, and 4 to 8 µl for carrying 0–800 µM MEHP in 1 ml growth medium. The morphology of cells before harvest is shown in (**B**). * and **: *P*<0.05 and *P*<0.01 vs. DMSO cells, *N*=3; scale bar=100 µm.

It is of interest to understand the toxic effects of MEHP on myoblasts before examining its physiological effects. To this end, various dosages of MEHP were applied to confluent myoblasts (CMB) for 48 h before cells were harvested for MTT assay. We found that cell viability was not affected by MEHP when it was less than 100 µM (Figure 1A). However, higher level MEHP triggered cell detachment and death. Besides, the morphology of cells treated with higher level of MEHP was also changed (Figure 1B and Supplementary Figure S1). These observations suggest that the toxic effect of MEHP less than 100 µM is tolerable to myoblasts for a certain period without immediate damage, although it might still be a stress to cells. Therefore, it suggests that this level of MEHP might be appropriate to observe its physiological effects on myogenic cell metabolism and organelle homeostasis.

### MEHP presents *in vitro* stage-specific effect on myogenic differentiation

**Figure 2:**
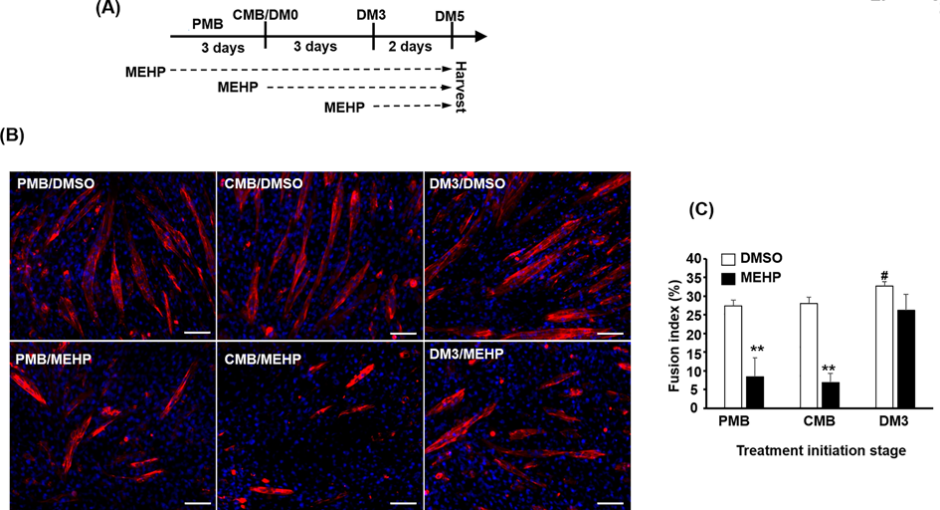
The effects of MEHP on myogenic differentiation C2C12 myoblasts were treated with 100 μM MEHP starting at PMB, CMB, and DM3 stages and all cells were harvested/photographed after 5 days in differentiation medium (DM5). The experimental process is shown in panel (**A**). (**B**) Multinucleated myotubes were stained with immunofluorescence using the antibody against myosin heavy chain (MHC). The fusion index (nuclei in myotubes/total nuclei) in percentage is shown in panel (**C**). **: *P*<0.01 vs. DMSO; #: *P*<0.05 vs. PMB, *N*=3; scale bar=100 µm.

To examine the possible effects of MEHP on myogenic differentiation *in vitro*, C2C12 myoblasts were treated with 100 μM MEHP starting at proliferating myoblast (PMB), confluent myoblast (CMB), or myotube (after 3 days in differentiation medium, DM3) stage and harvested on DM5 (Figure 2A). Morphologically, we found a stage-specific effect on myogenesis as myotube numbers and the fusion index (nuclei in MHC^+^ myotubes/total nuclei) was significantly reduced on myoblasts receiving MEHP treatment starting from PMB and CMB stages but not on those (DM3) treated after the differentiation process had been started (Figure 2B,C). These observations imply that MEHP might block the activation and function of early factors, such as *Myogenin* and *Mef2c*, for initiating myogenesis when myoblasts are still highly proliferating or stimulated by growth factors in the growth medium (GM). After differentiation has initiated by cell–cell contact and the withdrawal of growth factors, the anti-myogenesis effect of MEHP on these myogenic factors can be highly reduced as these factors have been either expressed or activated.

### MEHP profoundly interferes mitochondrial functions in myotubes

**Figure 3:**
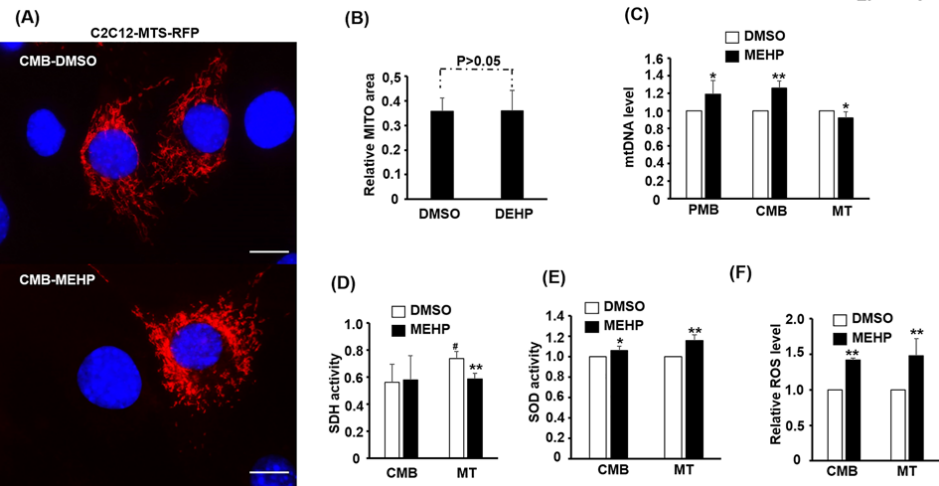
The effects of MEHP on mitochondria homeostasis and functions C2C12-MTS-RFP stable clone cells over-expressed with *MTS- RFP* at PMB stage were treated with 100 μM MEHP for 2 days and their morphology of RFP-labeled mitochondria is shown in panel (**A**). The MITO network areas of these cells were normalized with nuclear area and the relative MITO area is shown in (**B**); *N*=3. (**C–F**) C2C12 myoblasts at various stages were exposed to 100 μM MEHP for 2 days, and then their mtDNA levels (*N*=5), the activity of SDH (*N*=5) and SOD (*N*=4) enzymes, and ROS level (*N*=4) were determined. * and **: *P*<0.05 and *P*<0.01 vs. DMSO; #: *P*<0.05 vs. CMB; scale bar=25 µm

Mitochondria (MITO) are the major sites for oxidative metabolism, and its number and activity are both intimately correlated to cellular ability to metabolize carbohydrates and fatty acids efficiently. The morphology of MITO can be observed by the red fluorescence protein MTS-RFP targeting to MITO matrix (Figure 3A). In the stable clone C2C12-MTS-RFP carrying MTS-RFP expressing vector, MITO were found to form connecting filaments around the nucleus (Figure 3A). The size of MITO was measured by comparing its red fluorescence area in C2C12-MTS-RFP stable cells, and no significant difference in the MITO area between DMSO- and MEHP-treated cells was found (Figure 3B), suggesting that MEHP treatment has no effect on the size of MITO in myoblasts.

Mitochondria carry their own genomic DNA (mtDNA) and 13 protein-coding genes critical for oxidative phosphorylation (OXPHOS) are encoded in mtDNA. As the amount of mtDNA can be adjusted to meet physiological demands, the amount of mtDNA in each cell can be used as an index of mitochondrial function and number. We found that mtDNA was increased by MEHP in myoblasts at PMB and CMB stages, but was reduced by MEHP in myotubes (Figure 3C), suggesting a stage-specific effect.

It is of interest to observe if the organelle-specific enzyme activity is also affected by MEHP. Succinate dehydrogenase (SDH) is a mitochondrial enzyme involved in both critic acid cycle and OXHPOS, so its activity should be a good indicator of mitochondrial function. We found that SDH activity in C2C12 myoblasts was not affected by MEHP but that in the myotubes was reduced significantly by MEHP, indicating that the influence of MEHP on mitochondria should be more profound in myotubes (Figure 3D). Superoxide dismutase (SOD) activity is involved in the removal of reactive oxygen species (ROS) in the cells and is performed by several enzymes distributed in the mitochondria, peroxisomes, and cytoplasm. Total SOD activity, instead of organelle-specific, was measured and significantly higher activity was observed in both myoblasts and myotubes (Figure 3E).

The increased SOD activity is a hallmark of oxidative stress and it implies that MEHP can induce cellular oxidative stress either directly or indirectly. To verify this issue, total ROS was measured and we found very surprisingly high ROS in MEHP-treated cells, both myoblasts and myotubes (Figure 3F). The increased ROS in cells of different stages clearly indicates that MEHP stimulate ROS accumulation and the increased SOD activity might be secondary to the increased ROS level. Otherwise, the increased SOD activity should reduce ROS level instead of increasing it.

### MEHP promotes the formation of low efficient MITO in myoblasts

**Figure 4:**
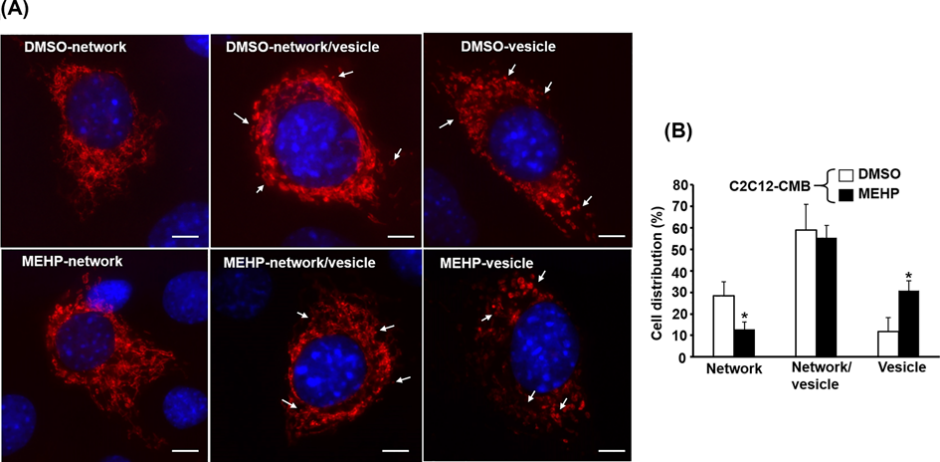
The mitochondrial network is affected by MEHP RFP-labeled MITO morphology in PMB stage C2C12-RFP-MTS cells treated with 100 μM MEHP for 2 days was photographed, and representative images of cells with filamentary network, mixture of network and vesicle, and vesicle MITO are shown in panel (**A**). The distribution of cells with various mitochondrial morphologies is shown in (**B**). Arrow: vesicular MITO. *: *P*<0.05 vs. DMSO, *N*=4; scale bar=10 µm.

It has been suggested that highly active MITO forms connecting network while low activity or degenerating MITO splits into vesicular ones [27,28], so the morphology of MITO is also a good indicator of their function. In CMB myoblasts, most cells contained filamentary and vesicular MITO but minor groups have only filamentary or vesicular MITO (Figure 4). However, the percentage of cells with filamentary MITO was reduced but those with vesicular MITO were increased in MEHP-treated myoblasts (Figure 4). Morphologically, these observations suggest that MITO function might be reduced drastically by MEHP in a minor group (approximately 20%) of cells resulting into the obvious change in their MITO organization. The MITO morphology of myotubes was also observed and all cells contain majorly filamentary network of MITO and the effect of MEHP on the morphology is negligible (data not shown).

**Figure 5:**
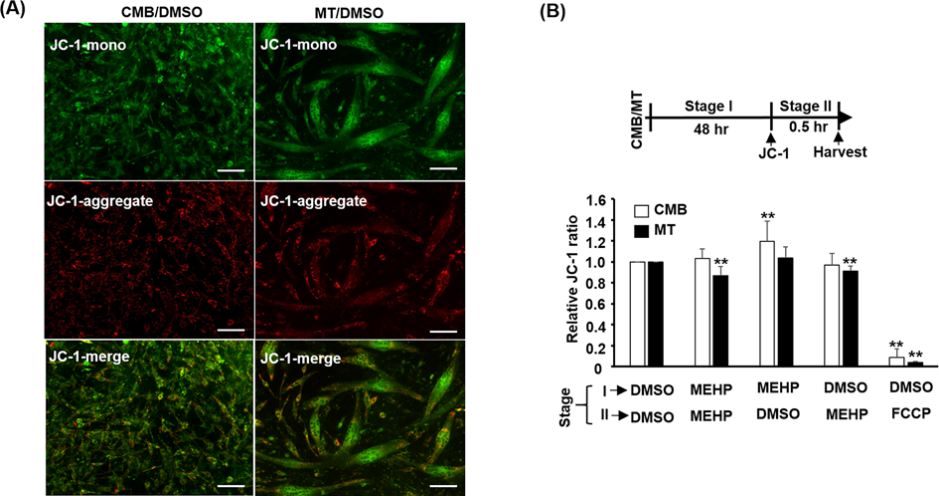
MITO membrane potential is marginally affected by MEHP (**A**) The mitochondrial potential of both myoblasts and myotubes treated with DMSO was revealed by JC-1 (2 µM) staining for 30 min before photographed under fluorescence microscope. JC-1 forms red (∼590 nm) aggregate in MITO but green (∼530 nm) monomer in cytoplasm, so the ratio of JC-1 staining can be used as a good indicator of Δ*ψ*_m._ (**B**) The MEHP treatment and the JC-1 staining/measurement periods were designated as stages I and II, respectively, in the top panel. Due to the quick change in mitochondrial potential, MEHP was included in either stages or both as indicated at the bottom. The intensity of JC-1 stain was measured in a fluorescence spectrophotometer and the relative ratio of absorbance 590/530 is calculated and shown in the bottom panel. The ratio in cells treated with DMSO at both stage was arbitrarily set as 1. FCCP (25 µM) treatment served as a control for JC-1 staining. **: *P*<0.01 vs. DMSO, *N*=4; scale bar=50 µm.

The increase in myoblasts with vesicular MITO raised the concern that MITO inner membrane potential (Δ*ψ*_m_) might be affected by MEHP. This issue was addressed by the MITO-specific staining of JC-1 that formed red (∼590 nm) aggregate in MITO but green (∼530 nm) monomer in cytoplasm, so the ratio of JC-1 staining can be used as a good indicator of Δ*ψ*_m_. The JC-1 red aggregates can be readily seen in both myoblasts and myotubes but no visible difference in staining was observable between cells treated with/without MEHP (Figure 5A and Supplementary Figure S2). The intensity of JC-1 stain was further measured in a fluorescence spectrophotometer and the relative ratio of absorbance 590/530 was calculated. To our surprise, myoblasts treated with MEHP at both treatment (stage I) and JC-1 staining (stage II) stages showed no change in Δ*ψ*_m_. But removal of MEHP during JC-1 staining increased Δ*ψ*_m_ marginally but significantly (Figure 5B). Including of MEHP at the staining stage has no effect on the Δ*ψ*_m_ of myoblasts. The Δ*ψ*_m_ of myotubes was reduced in cells treated with MEHP at both stages and those treated during staining stage, but not altered in cells treated with MEHP during stage I, suggesting the acute effect of MEHP on Δ*ψ*_m_, which is highly dynamic. Taken together, these observations suggest that the effect of MEHP on Δ*ψ*_m_ depolarization is very marginal and which is not the major cause of the compromised MITO functions observed in MEHP-treated cells.

### MEHP selectively regulates the expression of genes involved in oxidative metabolism

**Figure 6:**
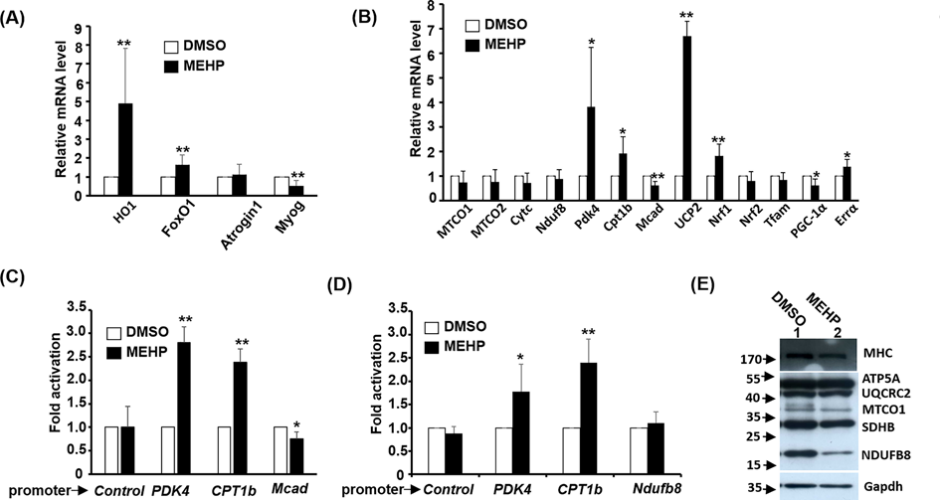
MEHP increases the expression of genes related to fatty acid metabolism Gene expression levels in C2C12 myotubes were exposed to MEHP (100 μM) for 2 days examined by qRT-PCR. The mRNA expression levels of genes related to muscle atrophy and ROS scavenge are shown in panel (**A**) and those for oxidative metabolism are shown in panel (**B**). (**C** and **D**) Luciferase-based reporters driven by promoter of the interested genes were transfected into C2C12 myoblasts and treated with/without MEHP (100 µM) for 2 days in DM before harvested for determining their luciferase activity. The vector pStable-luc served as a promoter-free control. Panels (C) and (D) were data of different co-authors performed at the different times during this study. (**E**) The protein levels of OXPHOS complex proteins and MHC in myotubes treated with/without MEHP were detected by Western blot. Gapdh was used as the loading control and numbers on the left side represents molecular weight in kilodalton. * and **: *P*<0.05 and *P*<0.01 vs. DMSO, *N*=4.

Our results described above suggest that MEHP should have profound effects on MITO homeostasis and functions; therefore, it is of interest to identify its target genes mediating its effects on oxidative metabolism performed in MITO. C2C12 myotubes were exposed to 100 μM MEHP for 2 days and the genes expression were examined by qRT-PCR. *HO-1* is a critical regulator of cellular ROS level and its expression was increased by MEHP (Figure 5A). *FoxO1* and *Atrogin-1* encode factors promoting SKM atrophy during fasting, and here we found only *FoxO1* expression was increased by MEHP significantly (Figure 6A), suggesting that MEHP might have minor effect on atrophy. The stage-specific effect of MEHP on myogenic differentiation (Figure 2C) suggests its targeting on myogenic genes, and we found that the expression level of the early myogenic marker, *Myogenin (Myog)*, was marginally suppressed by MEHP (Figure 6A).

The effects of MEHP on MITO, including mtDNA, enzyme activity, and morphology, described above prompted us to examine its influence on the expression of genes that are related to mitochondrial functions and ROS stress. The expression levels of *PDK4, Cpt1b, UCP2, Nrf1*, and *Errα* were significantly induced by MEHP while *Mcad* and *PGC1-α* expression were suppressed marginally by MEHP (Figure 6B). These factors are all involved in oxidative metabolism and their increased expression levels by MEHP suggesting a metabolic transition to oxidative metabolism.

The activation of *Cpt1b* and *PDK4* expression by MEHP is interesting as they are both involved in the metabolism of fatty acids (Figure 6C,D). Cpt1b is critical for the entry of fatty acids to mitochondria and PDK4 drives fatty acid oxidation by preventing pyruvate entry to TCA cycle. The activation of both gene promoters by MEHP was also observed (Figure 6C,D) and it suggests direct regulation of both genes by MEHP downstream effectors. The increased expression of *Cpt1b* and *PDK4* also implies a metabolic shift from glucose to fatty acid oxidation. However, the repression of *Mcad* expression and its promoter activity implies that fatty acid metabolism in mitochondria might be hindered by MEHP, suggesting a substitute, such as peroxisomes, for mitochondria in fatty acid metabolism might be involved.

### MEHP induces the protein degradation of OXPHOS complex

In the present study, we showed that MEHP affected mitochondrial functions and the gene expression related to oxidative metabolism. Oxidative phosphorylation is the most important pathway to produce ATP currency in mitochondria. This reaction is triggered by five OXPHOS complexes located at the inner membrane of mitochondria. The RNA levels of some key factors, MTCO1, MTCO2, cytochrome *C* (CytC), and Ndufb8, were examined but no significant change was triggered by MEHP (Figure 6B). We also examined the protein levels of key factors representing each OXPHOS complex after MEHP treatment and no significant effect was found in cells at CMB stage (data not shown); however, in myotubes, significantly lower levels of MTCO1 (complex IV) and Ndufb8 (complex I) were observed in MEHP-treated cells (Figure 6E). *MTCO1* is encoded in the MITO genome with very complicated transcriptional regulation in the MITO. In contrast, *Ndufb8* is a nuclear gene so we set out to analyze the regulation of *Ndufb8* promoter by MEHP but no significant effect can be observed (Figure 6D). Since the promoter activity and RNA level of Ndufb8 showed no significant difference after MEHP treatment (Figure 6B,D), it raises the possibility that MEHP may affect MTCO1 and Ndufb8 protein levels post-translationally.

**Figure 7:**
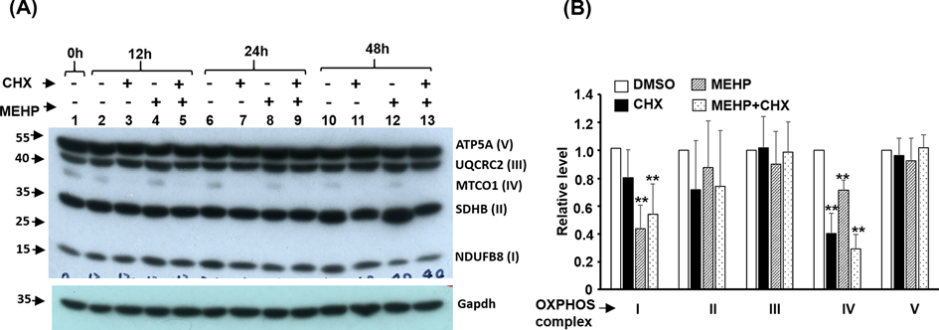
MEHP affects the protein expression of OXPHOS complex The protein levels of OXPHOS complex proteins C2C12 myotubes treated with MEHP (100 μM) and cycloheximide (CHX, 20 μg/ml) for 12, 24, and 48 h were determined with Western blot. A representative image is shown in panel (**A**), and the proteins levels at 48 h was quantitated and normalized with Gapdh and the relative levels are shown in panel (**B**). **: *P*<0.01 vs. DMSO, *N*=4.

The effect of MEHP on the stability of OXPHOS complex proteins was revealed by treating myotubes with Cycloheximide (CHX), a protein synthesis inhibitor. C2C12 myotubes were incubated with MEHP and/or CHX for various times and the protein levels of OXPHOS complexes were analyzed by Western blot. CHX showed a strong inhibitory effect on MTCO1 level as early as 12 h after treatment, suggesting that MTCO1 protein is unstable and its turnover rate might be high in myotubes. The effect of MEHP on other OXPHOS complex proteins was much weaker, even after 48 h, suggesting that these proteins are quite stable with long half-life in myotubes (Figure 7A). MTCO1 protein level was only slightly affected by MEHP and this effect was neither synergistic nor additive with CHX, implying that either they are acting on the same pathway or the minor effect of MEHP is covered by that of CHX. Ndufb8 level was not significantly reduced after treated with CHX for 48 h although it was significantly reduced by MEHP, suggesting that Ndufb8 protein is highly stable in myotubes Combined treatment with both compounds did not further reduced the protein levels of Ndufb8, which suggests that MEHP might reduce Ndufb8 protein level via enhanced protein degradation after translation in myotube.

### MEHP represses the oxidation of fatty acid by mitochondria

One of the metabolic functions of mitochondria is the β-oxidation of fatty acids to generate ATP currency through OXPHOS. The effects of MEHP on this function was examined by treating C2C12 myotubes with/without MEHP (100 μM) for 48 h (stage I) and then incubated with ^3^H labeled palmitic acid (PA) in the presence/absence of MEHP for 5 h (stage II). The oxidized PA in medium was extracted and determined by scintillation counter. Cells treated with MEHP only at the stage I showed enhanced PA β-oxidation (Figure 7). In sharp contrast, those treated with MEHP only at the stage II showed reduced PA β-oxidation. The effects of MEHP on myotubes treated at both stages were similar to those treated at stage II only. These observations suggest that MEHP is inhibitory to PA β-oxidation and the increased PA β-oxidation in cells treated only at stage I might be caused by the compensatory up-regulation of genes involved in β-oxidation (Figure 6B).

### Insulin-induced glucose uptake is reduced by MEHP

**Figure 8:**
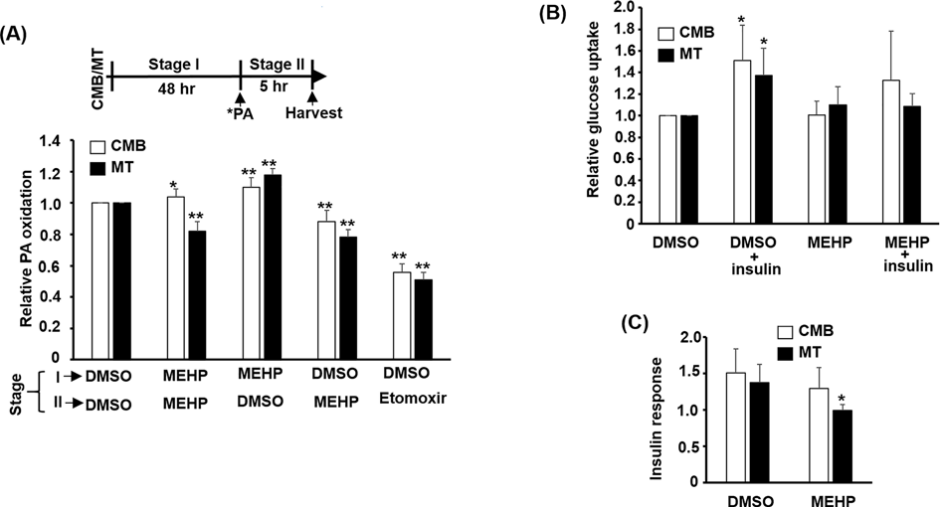
The effects of MEHP on the oxidation of palmitic acid and glucose uptake C2C12 myoblasts at CMB and MT stages were treated with MEHP (100 μM) for 2 days (stage I). Then, the relative oxidation levels of palmitic acid (**A**) and glucose uptake (**B** and **C**) were measured. Etomoxir (10 μM) is a CPT1b inhibitor and was used as a control for fatty acid entry to MITO. The top panel in (A) indicates stage I and II of the assay and the ^3^H-labeled palmitic acid (*PA) was added at the beginning of Stage II for 5 h. * and **: *P*<0.05 and *P*<0.01 vs. DMSO at both stage I and II, *N*=4. (B) Glucose uptake was performed with ^3^H-labeled 2-deoxy-glucose after serum starvation (4 h) and the insulin responses (insulin stimulated uptake/uptake in vehicle) are shown in panel (C). *: *P*<0.05 vs. DMSO only, *N*=4.

SKM is the largest glucose uptake and storage site, so it plays an important role in blood glucose clearance. It is of interest to know if MEHP affects SKM glucose uptake. Surprisingly, MEHP had no observable effect on basal glucose uptake (Figure 8B); however, MEHP significantly reduced insulin-induced glucose uptake in myotubes. Insulin response was calculated and it was repressed in myotubes (Figure 8C). These observations suggest that MEHP might specifically target insulin signaling transduction but not the glucose process as the basal glucose uptake was not affected by MEHP treatment.

## Discussion

Trunk and limb skeletal muscle (SKM) cells in vertebrate embryos are derived from *Pax3^+^/Pax7^+^*myogenic stem cells in somites, and these cells migrate from dermomyotome to myotome and limbs to form mature multinucleated myocytes [29]. The expression of either *MyoD* or *Myf5* confines them in the myogenic lineage and also drives them to become myoblasts. Upon the stimulation of differentiation signals, either MyoD or Myf5 starts inducing the expressing *Myogenin* and *Mef2c* that cooperatively drive the expression of contractile proteins, cell cycle exit, and formation of multinucleated myotubes [19]. C2C12 myoblasts express high level of *MyoD* and they start differentiation when cells are contacting each other under low mitogen condition (2% horse serum), recapitulating the differentiation process *in vivo*. Therefore, to understand the timing effects of MEHP on myogenic differentiation, it should be applied before (PMB) and after cell–cell contact (CMB) and after initiation of differentiation under low serum condition (DM3).

It has been shown that DEHP can reduced the expression of *MyoD* and partially inhibit terminal myogenic differentiation but only at very high dose, 1000 µg/ml, which is approximately 2.56 mM [30]. Here we found that MEHP, the immediate metabolite of DEHP, was highly toxic to myoblasts at level higher than 200 µM (Figure 1), so cells in all experiments were treated with 100 µM MEHP. Myoblasts treated at PMB and CMB stages showed serious defects in myotube formation but those received MEHP after DM3 showed only minor, but non-significant, effects on myogenesis. However, the expression levels of *Myogenin* and *Mef2c* were also reduced in DM3 treated myotubes (Figure 6A; data not shown), implying that MEHP is highly inhibitory to the activation of *Myogenin* and *Mef2c* expression by MyoD or other upstream activators. The discrepancy between ours and previous study suggests that myoblasts are highly sensitive to MEHP as compared with DEHP. In the future, it will be interesting to dissect how the transactivational activity of MyoD was affected by MEHP.

Mitochondria are highly dynamic and their number is regulated by the balance between biogenesis and MITO-specific autophagy (mitophagy) to meet physiological demand, such as exercise, in SKM. Oxidative exercise induces MITO proliferation to promote oxidative respiration, which in turn will increase the number of type I myotubes in SKM while sedentary life or disuse antagonizes this process [31]. The fusion and fission of existing MITO determine the morphology of MITO in the cell, fission triggers formation of vesicular MITO and fusion derives filamentary network. Fusion is protective to mtDNA as loss of fusion results in increased mutation rate and genome loss, especially in SKM [32]. In the present study, although most (approximately 55%) myoblasts were in homeostasis, a subgroup (approximately 20%) of cells had lost their filamentary network after MEHP treatment (Figure 4), implying susceptible to more mtDNA mutation and defects in MITO functions. The reduced SDH activity and surged ROS level in MEHP treated cells (Figure 3D,F) have soundly proved this notion.

Both fusion and fission are mediated by members of the dynamin GTPase-related protein (DRP) family. DRP proteins, Mfn1 and Mfn2, on the outer membrane and Opa1 on the inner membrane work together to promote MITO fusion. During fission, DRP effectors MDv1, Mff, MiD49, and MiD51 recruit DRP1 to outer membrane to assemble into a helical structure that wrap around MITO to trigger fission. Enhanced fusion creates MITO network that are higher in membrane potential (Δ*ψ*_m_) and ATP production efficiency, on the contrary, fission generates more efficiency poor MITO (reviewed in [27]). It will be interesting to know that if MEHP enhances the expression and function of fission factors but represses that of fusion factors.

The increase in low efficiency MITO might also reflect slow removal of old MITO. The quality of MITO is regulated by several pathways and severely damaged MITO are removed via mitophagy, in which MITO are wrapped in double-membrane vesicles, known as autophagosomes, and are delivered to lysosomes for degradation. MITO damage stabilizes PINK1 kinase on the outer membrane that phosphorylated Mfn2 to serve as a receptor for the cytosolic E3-ubiquitin ligase Parkin. Parkin interact with several autophagy-related proteins to promote MITO removal [33].

The increased expression of *PDK4* and *CPT1b* in myotubes by MEHP (Figure 6) suggests that glucose metabolism via TCA cycle has been blocked but fatty acid metabolism entry into mitochondria for β-oxidation has been increased. The reduced glucose uptake confirmed the slowdown of glucose metabolism (Figure 8B,C); however, it was intriguing to find that PA β-oxidation was increased by MEHP in cells treated at stage I only but reduced in cells treated at stage II (Figure 8A). The stage II-specific effect on PA β-oxidation implies that MEHP should function as an inhibitor of fatty acid metabolism, either in the transportation or oxidation process. The increased *PDK4* and *CPT1b* might reflect a compensatory response to counter this inhibition, which resulted in a false image of increased fatty acid metabolism in cells treated only at stage I (Figure 8A). Therefore, our observations suggest that both fatty acid and glucose metabolisms are inhibited by MEHP in SKM. As MEHP can function as a PPAR ligand, it is supposed to activate oxidative metabolism via PGC-1α coactivated pathways [34]. The inhibition of oxidative metabolism by MEHP was contradictory to its function as a PPAR ligand, suggesting that other pathways might be involved in its action and similar observation has also been found in *PPARα* knockout mice [13]. Besides, DEHP showed tissue-specific effects and it induced serious accumulation of pyruvate and lactate in SKM but reduction of both metabolites in liver [11], suggesting repression of glucose oxidative metabolism in SKM as we observed in the present study.

Induction of ROS by MEHP/DEHP has been observed in many different cell types, including liver, testicular, ovarian, and endothelial [35–38]. In some studies, association of MITO membrane potential dysfunction was found to accompany ROS surge, but not in all case. In the present study, ROS surge (Figure 3F) was not associated with MITO membrane potential loss as JC-1 staining showed only marginal difference between control and MEHP-treated cells, especially in myotubes (Figure 5). In most cell types studied, the surged ROS usually associated with apoptotic cell death via the MITO-dependent pathway, a phenomenon not observed in the present study. It seems that myoblasts and myotubes have higher tolerance to MEHP than other cells types as the dosage (100 µM) used here can cause large amount of cell death in other cell types but not in SKM cells. Nevertheless, the pathways leading to increased ROS surge are poorly defined in most studies. Furthermore, it is unclear whether ROS is the cause or the effect of metabolic dysfunctions, such as reduced glucose uptake and fatty acid oxidation. In the future, reducing agents, such as N-Acetylcysteine and ascorbic acid, should be included in the MEHP treatment to neutralize ROS for analyzing the cause-or-effect role of ROS on metabolic disorder observed in the present study.

The ROS surge prompted us to examine the expression of OXPHOS complex proteins and the drastic reduction in Ndufb8 protein level but not in mRNA (Figures 6 and 7) suggests a posttranslational protein stability regulation. Usually, OXPHOS complex I and III dysfunction is the major cause of MITO-derived ROS surge, and the reduced Ndufb8 protein stability suggests that dysfunction of complex I might be the cause of ROS surge in MEHP-treated cells. Currently, we are establishing an inducible over-expression system for compensating the reduced Ndufb8 protein level to confirm its role in ROS surge.

Among all the physiological parameters (not including gene expression) examined in the present study, ROS surge showed the greatest change and it is frequently associated with DEHP/MEHP treatment (as discussed above); therefore, it might be the major cause responding for most, if not all, of the repressed MITO functions and homeostasis observed in the present study. Besides, all other parameters measured here can be induced by the surged ROS [39]. However, as SKM cells are designed for contraction to exercise, a process that generate a lot of ROS, they can efficiently adapt to an environment of high ROS so the ROS-induced physiological changes are much minor as compared with cells of other tissues. The post-translational change in Nduf8 protein level is a novel observation not associated with DEHP/MEHP treatment before, so it might be a SKM-specific effect. To explain the underlying mechanism, we hypothesize that MEHP triggered Ndufb8 protein reduction in SKM, which consequently induced dysfunction of the OXPHOS complex I and then ROS surged. Further study on cells over-expressed with Ndufb8 by an inducible system should provide answer and evidence to our hypothesis.

## Supplementary Material

Supplementary Figures S1-S2 and Tables S1-S2

## Abbreviations

CMB: confluent myoblasts
DEHP: Di (2-ethylhexyl) phthalate
MEHP: mono(2-ethylhexyl) phthalate
MITO: mitochondria
mtDNA: MITO DNA
OXPHOS: oxidative phosphorylation system
PMB: proliferating myoblasts
PVC: polyvinyl chloride
ROS: reactive oxygen species
SKM: skeletal muscle
