# Supplementary material for: MEHP interferes with mitochondrial functions and homeostasis in skeletal muscle cells
Source: Biosci Rep. 2020 Apr 17;40(4):BSR20194404. doi: 10.1042/BSR20194404 (PMC7167251; doi:10.1042/BSR20194404)
Supplement: Supplementary Figures S1-S2 and Tables S1-S2 [file BSR-2019-4404_supp.pdf]

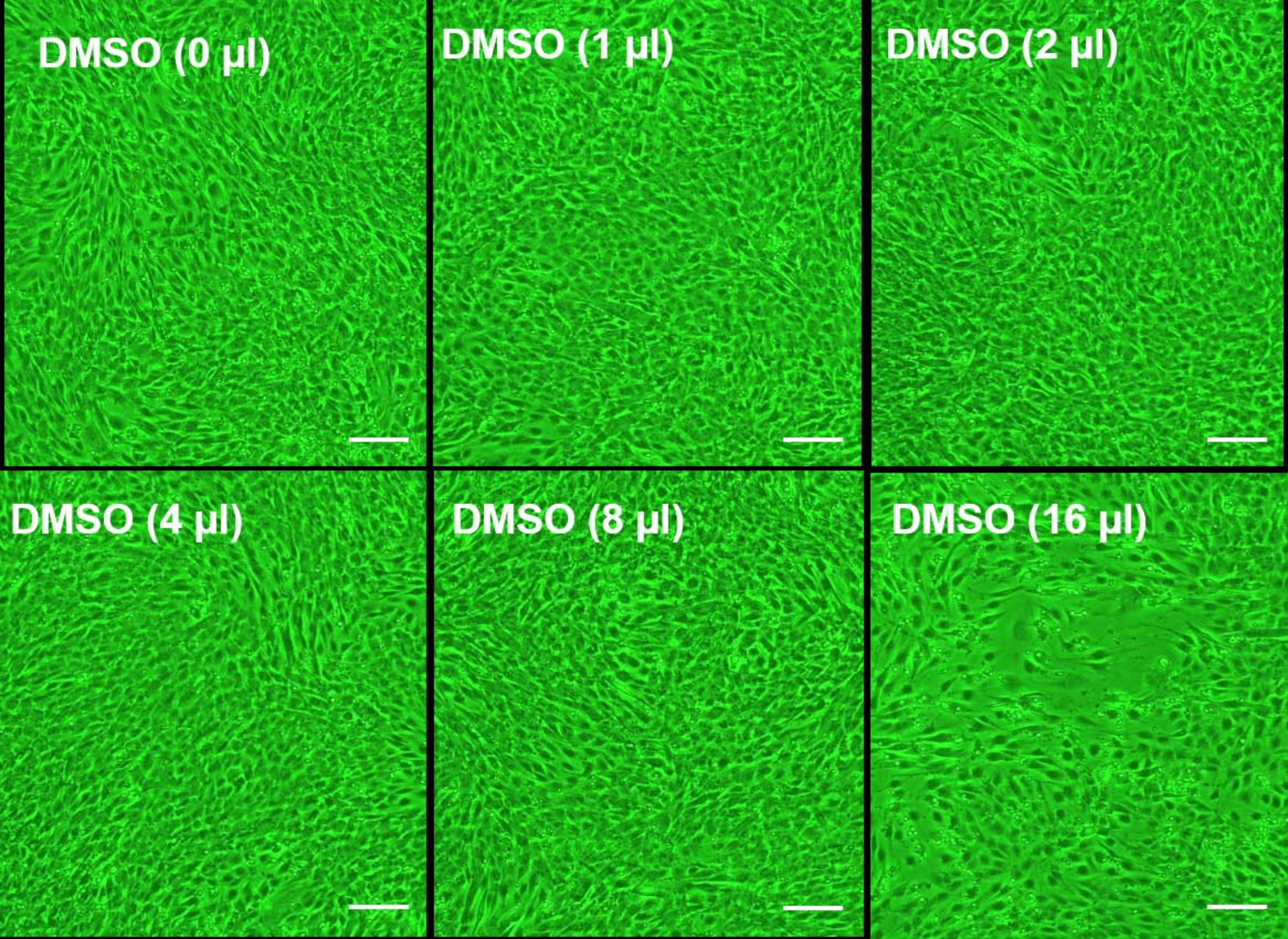
DMSO (0 µl)
DMSO (1 µl)
DMSO (2 µl)
DMSO (4 µl)
DMSO (8 µl)
DMSO (16 µl)

Supplementary figure 1

The representative images of myoblasts treated with various amount of DMSO as negative control of MEHP treatment. The amount of DMSO (μl/ml) added is indicated and cells were photographed after 2 days in treatment. Scale bar=100 μm

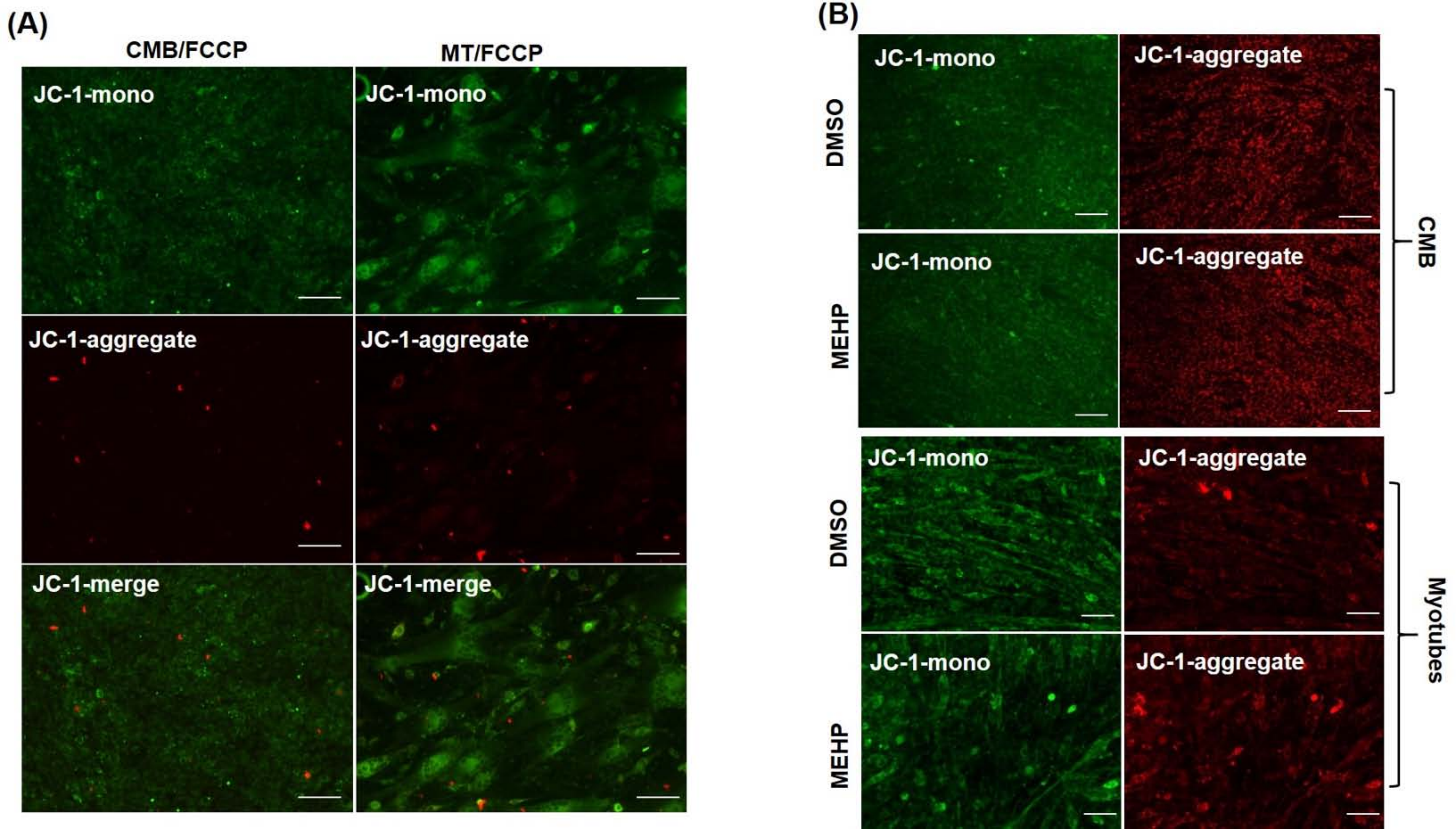
(A)
CMB/FCCP
MT/FCCP
JC-1-mono
JC-1-mono
JC-1-aggregate
JC-1-aggregate
JC-1-merge
JC-1-merge
(B)
DMSO
JC-1-mono
JC-1-aggregate
MEHP
JC-1-mono
JC-1-aggregate
CMB
DMSO
JC-1-mono
JC-1-aggregate
MEHP
JC-1-mono
JC-1-aggregate
Myotubes

Supplementary figure 2

(A) The representative images of myoblasts and myotubes treated with FCCP (25 μM) as a control of JC-1 staining. FCCP depolarized the mitochondrial potential so the JC-1 aggregate (red) disappeared as compared with cells without this treatment. Scale bar=50 μm. (B) Representative JC-1-staining images of myoblasts and myotubes treated with/without MEHP. JC-1 that formed red (~590 nm) aggregate in MITO but green (~530 nm) monomer in cytoplasm. Scale bar=100 μm.

Supplementary table I: Primer sets used in the quantitative real-time PCR.

NCU code	Gene name	Primer Sequence[a]	Amplicon size (bp)
029088<br>029089	*PGC-1α*	FP: 5'-CGTGACCACTGACAACGAG-3'<br>RP: 5'-CTGCATGGTTCTGAGTG-3'	164
000053<br>000054	*MTCO1*	FP: 5'- CGCAATTCCTACCGGTGTCA-3'<br>RP: 5'- TGAAGCACGATGTCAAGGGA-3'	168
001056<br>001057	*MTCO2*	FP: 5'-AACCGAGTCGTTCTGCCAATAG-3'<br>RP: 5'-ACCCTGGTCGGTTTGATGTTAC-3	154
000047<br>000048	*Nrf1*	FP:5'-CAAGTCCAGCAGGTCCATGT -3'<br>RP:5'-ATGAGGCCGTTTCCGTTTCT -3'	219
063005<br>063006	*Nrf-2*	FP: 5'-AGTTCACCGGGGAACAGAAC-3'<br>RP: 5'- CGCAACCAACTCAGGCTGAT-3'	150
063003<br>063004	*Errα*	FP: 5'- ACGCTCTGGTGTCGCATCTG-3'<br>RP: 5'- CTGACATCTGGTCAGACAGT -3'	195
037019<br>037020	*Tfam*	FP: 5'- ATGGCGCTGTTCCGGG-3'<br>RP: 5'-GATAGCTACCCATGCTGGA -3'	145
041003<br>041004	*HO1*	FP: 5'-GCCCCAGGATTTGTCTGA-3'<br>RP: 5'-CAGGGCCGTGTAGATATGGT-3'	157
005027<br>035006	*Atrogin-1*	FP: 5'-CAGCCTGAACTACGACGTC-3'<br>RP: 5'-GCTTCCCCCAAAGTACAGTA-3'	150
026046<br>026047	*FoxO1*	FP: 5'-CGCCGCAACGCGTGGGGCAACC-3'<br>RP: 5'-CCCTCTGGATTGAGCATCCACC-3'	263
006012<br>006013	*Myogenin*	FR: 5'-CCAGTGAATGCAACTCCCACAGC-3'<br>RP: 5'-AGACATATCCTCCACCGTGA-3'	166
063009<br>063010	*Ndufb8*	FP: 5'-GAACTGGGGTGAACCGATAC-3'<br>FP: 5'-GCAGCACGTAGAGGGAAGGA-3'	225
029112<br>029113	*Cytochrome c*	FP: 5'-TCTCCACGGTCTGTTCGG-3'<br>RP: 5'-GGGTATCCTCTCCCCAGGTG-3'	98

029067<br>029068	*PDK4*	FP: 5’-CGGTCGAGCATCAAGAAAAC-3’<br>RP: 5’-AACCAAAACCAGCCAAA-3’	207
029029<br>029030	*UCP2*	FP: 5’-GGATACCGCCAAGGTCCG-3’<br>RP: 5’-ATTGTAGAGGCTGCGTGG-3’	139
029116<br>029117	*Mcad*	FP: 5’-AGCTGATTGGCAATGTCTCC-3’<br>RP: 5’-GATCGCAATGGGTGCTTTTG-3’	291
001042<br>001043	*Gapdh*	FP：5’-CCTCTGGAAAGCTGTGGCGT-3’<br>RP：5’-TTGGCAGGTTTCTCCAGGCG-3’	190
006001<br>006002	*M36b4*	FP：5’-GGCAGCATTTATAACCCTGAAGTG-3’<br>RP：5’-CGGACACCCTCCAGAAAGC-3’	73
033034<br>033035	*MyoD promoter*	FP: 5'-CGACGCGTCCTCCTCTGTGCTGCCTTA-3’<br>RP: 5'-CGACGCGTCTTTCAGGCTGTGCAC-3	395
021045<br>000003	*Oct4 promoter*	FP: 5'-CGACGCGTCGAGGGTGCAGTGCCAACAG-3’<br>RP: 5'-GCGACGCGTGGGCACCCCGAGCCG GGGG-3	348
037029<br>037030	*Cytochrome b*	FP: 5'-TCCTATCAGCCATCCCA-3’<br>RP: 5'-GTTTGATCCTGTTTCGTGG-3’	173

[a]FP: forward primer; RP: reverse primer

Supplementary table II: Primer sets used in the PCR for cloning.

NCU code	Gene name	Primer Sequence[a]	Amplicon size (bp)
041060<br>041061	*MTS-RFP*	5’- GAGCTAGCATGTCCGTC -3’<br>5’- TTAGCGACCGGTCCACCGT -3’	903
063013<br>063014	*Ndufb8*<br>*promoter*	FP: 5’-CAGCCACATCCACGGGACTA-3’<br>RP: 5’-CCCAAGCTTGGGCCTCCATCCCGAAGCAGTAA-3’	2650
063011<br>063012	*Ndufb8 CDS*	FP: 5’-CGGAGAAGGTGAAGATGGCT-3’<br>RP: 5’-GCCCATCAAGCCTCCTCAGA-3’	590

[a]FP: forward primer; RP: reverse primer
